# The Influence of a Fire at an Illegal Landfill in Southern Poland on the Formation of Toxic Compounds and Their Impact on the Natural Environment

**DOI:** 10.3390/ijerph192013613

**Published:** 2022-10-20

**Authors:** Wojciech Rykała, Monika J. Fabiańska, Dominika Dąbrowska

**Affiliations:** Faculty of Natural Sciences, Institute of Earth Sciences, University of Silesia in Katowice, Będzińska 60 Str., 41-200 Sosnowiec, Poland

**Keywords:** landfill, fire, contamination, PAHs, leachate, gas chromatography (GC-MS)

## Abstract

Landfill fires pose a real threat to the environment as they cause the migration of pollutants to the atmosphere and water sources. A greater risk is observed in the case of wild landfills, which do not have adequate isolation from the ground. The aim of this article is to present the results of studies on the toxicity of waste from a fire in a landfill in Trzebinia (southern Poland). Both soil and waste samples were investigated. The samples were analyzed using the GC-MS method and the leachates using ICP-OES. A total of 32 samples of incinerated waste and soil were collected. The organic compounds included naphthalene, fluorene, phenanthrene, anthracene, acenaphthene, acenaphthylene, fluoranthene, pyrene, benzo (c) phenanthrene, benzo (a) anthracene, chrysene, benzo (ghi) fluoranthene, benzo (b + k) fluoranthene, benzo (a) fluoranthene, benzo (c) fluoranthene, benzo (a) pyrene, benzo (e) pyrene, perylene, indeno[1,2,3-cd] pyrene, benzo (ghi) perylene, and dibenzo (a + h) anthracene. Among the inorganic parameters, sulfates, chlorides, arsenic, boron, cadmium, copper, lead, and zinc were taken into account. Phenanthrene reached values exceeding 33 mg/L. Fluoranthene dominated in most of the samples. Sulfates and chlorides were present in the samples in concentrations exceeding 400 and 50 mg/L, respectively. Compounds contained in burnt waste may have a negative impact on soil and water health safety. Therefore, it is important to conduct research and counteract the negative effects of waste fires.

## 1. Introduction

The problem of landfill fires is becoming a more common and dangerous phenomenon worldwide every year [1,2,3,4]. Wild landfills occur most frequently, for example, on forest margins, in ditches, and on the peripheries of inhabited areas [5]. Since the oil, solvents, fuel, rusted metal, and batteries contained within them can cause severe environmental damage, they must be properly disposed of, which is expensive [6,7].

In 2012, the global generation of municipal solid waste (MSW) reached approximately 1.3 billion tons per year [8]. Although illegal dumping occurs for various reasons, one is thought to be the shortage of proper waste treatment facilities [9], while another could be the desire to dispose of waste cheaply. The problems with waste storage in Poland are influenced by economic and political factors [10].

Every day, about 10–30% of the MSW generated in metro cities and 30–50% in smaller cities and towns is left uncollected [11]. Another problem may be the low level of waste recycling. For example, the rate of recycling in Malaysia is as low as 15%, far behind developed countries such as Singapore, Germany, and South Korea, in which the annual rate is in the range of 50% to 75% [12].

One of the dangers that can arise from illegal landfills is fire, which can be caused by deliberate arson or spontaneous combustion resulting from the chemical reactions of waste, reactive materials, failure of landfill gas systems, smoking or sparks, landfill equipment, lightning strikes, or heated waste [13]. The main cause of fire is methane, as it is highly flammable [14]. It makes up about 40–60% of landfill gas. Carbon dioxide accounts for about the same amount, and the remainder is composed of nitrogen, oxygen, hydrogen, ammonia, sulphides, and other gases [15]. The amount of methane generated and migrating through a MSW landfill can lead to the increased intensity of the fire [16,17]. Over the years, according to the State Fire Service, there has been a consistently high number of landfill fires, which sometimes stayed active for days [18]. 

Higher temperature fires can cause the breakdown of volatile compounds which emit dense black smoke [19]. Such uncontrolled burning carries the risk of spreading rapidly beyond the landfill’s perimeter and can lead to air pollution. In the aftermath, dense smoke can travel for several kilometers, threatening nearby residents. The consequences of a landfill fire can also produce a potential chemical hazard. Burnt waste on unprotected soil may lead to the physical and chemical degradation of the soil [20], which can cause it to migrate to aquifers [21]. 

An example of the most hazardous solid waste that can be found in wild landfills is used car tires. Approximately 240 million vehicle tires are discarded worldwide every year [22]. Only around 25% of the discarded tires are reprocessed/reused, while the remaining ones end up in landfills or illegal dumps [23]. These man-made products mainly consist of styrene-butadiene, poly (butadiene), bromated butyl rubber, carbon black, extender oils, nylon, and steel wire [24]. In open fires, tire emissions reflect their chemical composition, which is 50% natural or synthetic rubber by weight, 25% black carbon, 10% metal (mostly in the steel belt), 1% sulfur, 1% zinc oxide, and trace quantities of other materials [25]. 

After being landfilled, waste materials are subjected to a series of physicochemical and biological transformations, provided to create leachate [26]. The leachate composition varies among landfills depending on the type of waste buried, degradation stage, climate conditions, characteristics of the landfill site, socioeconomic factors, and landfill technology [27,28]. 

Heavy metals and polycyclic aromatic hydrocarbons (PAHs) pose a potential threat to the environment and nearby residents. PAHs have been reported to have carcinogenic properties, and 16 PAHs have been listed as priority pollutants by the US Environmental Protection Agency [29]. There is the potential for them to be generated in situ in municipal landfill fires [30,31,32].

Environmental aspects related to the impact of potential landfill fires have only been addressed in several scientific articles. In the work of Øygard et al. [4], landfill leachate collected during and after an accidental landfill fire was tested. Levels of selected metals and compounds were determined compared to compounds present in the leachate under normal conditions. Research conducted by Raudonyte-Svirbutaviciene et al. [33] focused on soil contaminated with PAHs and heavy metals after a tire landfill fire in Lithuania. Escobar-Arnanz et al. [34] used two-dimensional gas chromatography-time-of-flight mass spectrometry to analyze soil samples from an uncontrolled tire dump fire. In another work by Rim-Rukeh [35], five landfills in Nigeria, where fires are most common, were tested for air pollution. Precipitation leachate that washed away long-term burnt waste after a previous landfill fire was analyzed in Romania [36].

PAHs are among the pollutants hazardous to human health and have been included in the list of hazardous substances, with important factors being the quantity of the substance, the duration of exposure, the route of exposure, and the effects of other chemicals to which the body is exposed. PAHs can penetrate the body via the respiratory and digestive systems, and by direct skin contact with particular substances such as soot and tar [37]. As persistent compounds, PAHs do not have a carcinogenic or mutagenic effect themselves, but their metabolites do [38]. To determine the harmfulness of PAHs, indicators of the toxicity of individual PAHs were introduced. Benzo(a)pyrene (BaP) is used as a reference substance, and the carcinogenic strength of Toxicity Equivalence Factor (TEF) of other compounds is calculated against BaP. The toxicity equivalent RTBaP Toxic Equivalent (TEQ) is the sum of the concentrations of individual PAHs and their relative toxicity factors [39], and it is calculated using Formula (1):

RTBaP = 0.001 × [Na] + 0.001 × [Acy] + 0.001 × [Ace] + 0.001 × [Fl] + 0.001 × [Fen] + 0.01 × [An] + 0.001 × [Flu] + 0.001 × [Pir] + 0.1 × [BaA] + 0.01 × [Ch] + 0.1 × [BbF] + 0.1 × [BkF] + 1 × [BaP] +5 × [DBA] + 0.1 × [BghiP] + 0.1 × [IP] (1)

A TEF value of 0 means that the compound is not carcinogenic. In a scientific paper [40], the calculation of mutagenicity equivalent (MEQ) and carcinogenicity (TCDD-TEQ) was suggested in order to assess the harmfulness of PAHs. The mutagenicity equivalent is given as the sum of the concentrations of the individual PAHs and their relative mutagenicity factors:

MEQ = 0.00056 × [Acy] + 0.082 × [BaA] + 0.017 × [Ch] + 0.25 × [BbF] + 0.11 × [BkF] + 1 × [BaP] + 0.31 × [IP] + 0.29 × [DBA] + 0.19 × [BghiP](2)

Carcinogenicity is equivalent to the sum of the concentrations of individual PAHs and their relative carcinogenicity coefficients:

TCDD-TEQ = 0.000025 × BaA + 0.00020 × [Ch] + 0.000354 × [BaP] + 0.00110 × [IP] + 0.00203 × [DBA] + 0.00253 × [BbF] + 0.00487 × [BkF] (3)

The harmfulness of PAHs is also expressed as the ratio of PAHs classified as carcinogenic to the sum of all labelled PAHs:

ΣPAH_carc_/ΣPAH = ([BaA] + [BaP] + [BbF] + [BkF] + [Ch] + [DBA] + [IP])/([SWWA]) (4)

The closer the value of the ratio ΣPAH_carc_/ΣPAH is to 1, the greater the risk posed to the population.

The purpose of this study is to investigate the toxic substances generated during a wild landfill fire. Such research has not yet been performed in Poland. Moreover, in the world’s literature, the problem has not been sufficiently addressed, with such key information missing as the level of contaminants produced, their migration extent, and time present in the soil. A wildfire that occurred in the town of Trzebinia (Poland) (2016 y.) was examined to assess its potential threat to the nearby natural environment. The soil incinerated waste and the leachate generated by washing incinerated waste from the landfill were tested for compounds and elements hazardous to health and the environment. Compounds detected in all samples were identified, and their abundance and profiles were recalculated using appropriate quantitative methods. 

## 2. Materials and Methods

### 2.1. Study Area

The wild dump (28,000 m^2^) is located in the southern part of the town of Trzebinia (southern Poland). It is situated in a tree-lined space. To the west, there is the Trzebinia Penitentiary (approx. 130 m), to the north is an industrial area (approx. 284 m). The types of solid waste stored in the landfill included used tires, black rubber, plastic packaging, and textiles, among others.

**Figure 1:**
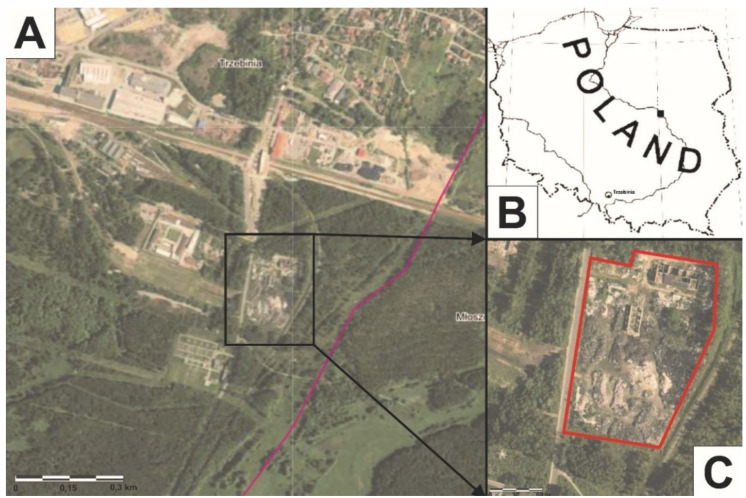
(**A**–**C**) Wild landfill fire location in Trzebinia on Słowackiego Street.

Between 26 and 27 May 2018, a fire occurred at the wild landfill, at around 1:30 am (Figure 1). The fire spread at a swift pace, which made the firefighting operation difficult. The fire was extinguished by pouring water on it, and then the area was covered with firefighting foam to protect the nearby forest. For at least two days, the smell of the fire could be felt within a radius of 6–7 km. Children and the elderly living in the area were advised to stay inside their homes. No one was injured or killed in the fire. 

According to reports from nearby authorities, the landfill described had been unused since 2013. The owner was required to dispose of the waste stored there. However, for unexplained reasons, he abandoned the facility. Since then, waste has been a never-ending problem for the town government, nearby residents, and the natural environment.

The ensuing fire created a huge, dark cloud of smoke that spread to nearby neighborhoods. This garbage created very poisonous and dense fumes that could, among other things, impede firefighting operations in the area. According to data provided by “Airlyeu” on the fire, in Chrzanów, the PM 2.5 and PM 10 standards were exceeded by 336% and 260%, respectively. 

**Figure 2:**
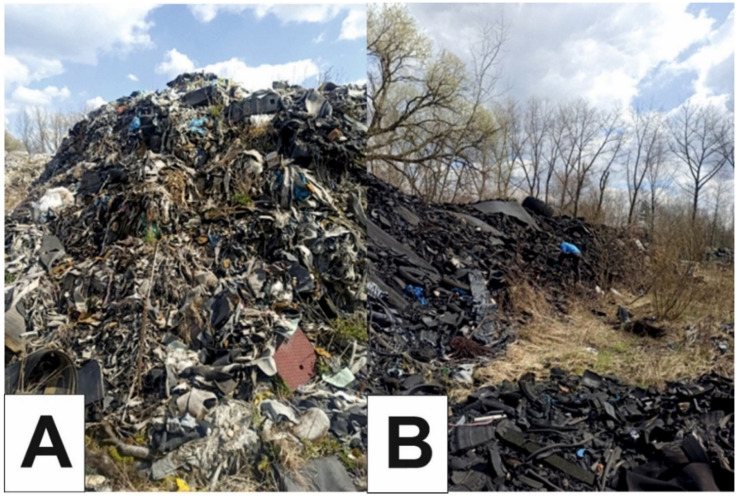
(**A**,**B**) The present site of the burnt wild waste dump in Trzebinia on Słowackiego Street (April 2021).

The charred, leftover waste remains uncovered at all times, unprotected from the elements (Figure 2A,B). During rainfall, waste that was altered by the fire is systematically washed away. The resulting leachate then infiltrates deep into a nearby aquifer, creating a dangerous “pollution cloud”.

### 2.2. Sampling

During two days of sampling in the morning hours of 24 April 2021, and 24 November 2021, a total of 32 soil and burnt solid waste samples were collected from the study area of the wild landfill in Trzebinia (Table 1). All samples included in the study were collected at an appropriate distance using a pre-planned route.

Samples of approximately 20 g of surface soil and waste were collected with a steel spatula in glass jars and then closed tightly and transported to the laboratory. Next, the soil samples were air-dried in a clean laboratory room. 

### 2.3. Sample Preparation and Characterization

#### 2.3.1. Sample Preparation for Laboratory Analysis

**Figure A1:**
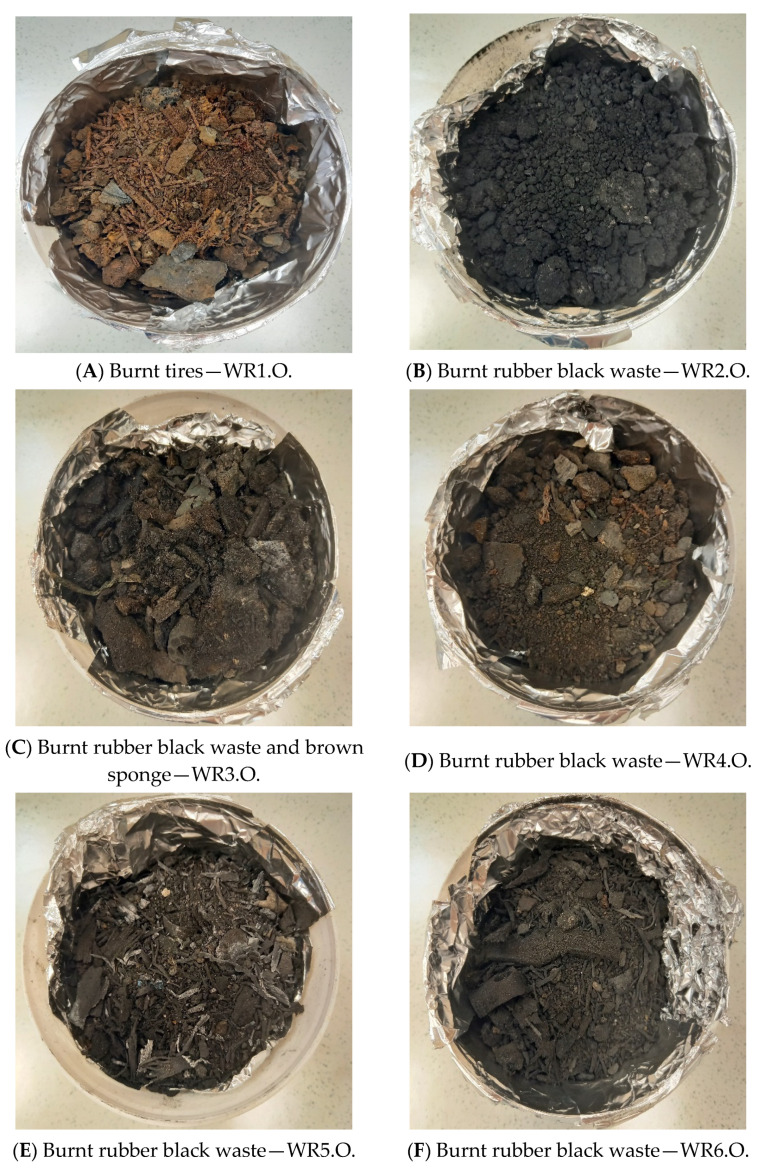

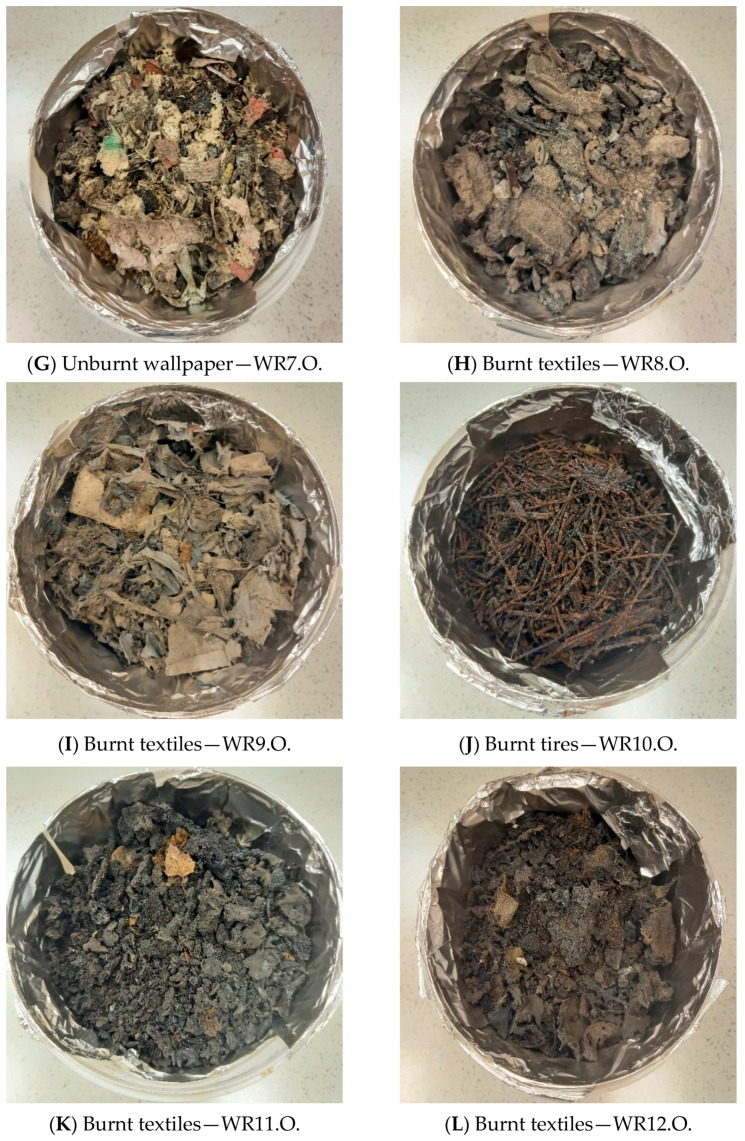
(**A**–**L**) Presentation of 12 solid waste samples subjected to the leaching process.

The 12 waste samples (Appendix A Figure A1A–L) collected on 24 November 2021, were prepared for transport to the i2 Analytical Limited Sp. z o.o. Pionierów 39 Str. accredited laboratory in Ruda Śląska. The objective was to perform specific tests on the content of leachate generated from the aforementioned waste. The laboratory was commissioned to test the contents for inorganic compounds: chlorides, sulfates, and heavy metals: boron, arsenic, cadmium, copper, lead, mercury, and zinc.

Before being sent to the laboratory, the waste samples were dried, weighed, flooded with demineralized water, and then washed according to the PN-EN 12457-2 Norm. The resulting leachates were filtered and transported to an accredited laboratory in glass containers at room temperature.

Chlorides were analyzed using the colorimetric method using a Discrete Analyzer, while the other ordered compounds were analyzed using the Inductively Coupled Plasma Optical Emission Spectrometry (ICP-OES) technique. All analyses were performed using ISO 17025 accreditation and the procedure L039-PL.

#### 2.3.2. Sample Preparation for GC-MS

Appropriately dried, cataloged, and aged transformed solid waste samples were extracted with dichloromethane in an ultrasonic bath for 15 min at 30 °C. The extracts were filtered, the solvent evaporated, and the extract yields calculated. All samples were derivatized (silanized) prior to gas chromatography-mass spectrometry (GC-MS) analysis. 

#### 2.3.3. Gas Chromatography-Mass Spectrometry (GC-MS)

The GC-MS analyses were carried out using an Agilent Technologies 7890A gas analyzer chromatograph and Agilent 5975C network mass spectrometer with the triple-axis detector system at the Department of Natural Sciences, Sosnowiec, Poland, using helium (6.0 Grade) as the carrier gas at a constant flow rate of 2.6 mL/min. Separation was obtained with a J&W HP5-MS (60 m × 0.32 mm i.d., 0.25 μm film thickness) fused silica capillary column coated with a chemically bonded phase (5% phenyl, 95% methylsiloxane), for which the GC oven temperature was programmed from 45 °C (1 min) to 100 °C at 20 °C/min, then to 300 °C (hold 60 min) at 3 °C/min, with a solvent delay of 10 min [41]. Mass spectra were recorded from 45 to 550 da (0–40 min) and 50–700 da (>40 min) electron impact mode, with an ionization energy of 70 eV. Data were acquired in full scan mode and processed with the Hewlett Packard Chemstation software. The compounds were identified by their mass spectra, and a comparison of peak retention times with those of standard compounds was carried out, as well as an interpretation of MS fragmentation patterns, and literature data [42].

Peaks were integrated manually. Quantitative analysis was performed based on the 5-point calibration curves for the analytical standards. The linear correlation between the peak areas and PAH concentrations was checked within the range of 0.10–10 μg/mL, with correlation coefficient values within the range of 0.997–0.998. For quality assurance and quality control (QA/QC), the analysis of each sample series was accompanied by the analysis of a blank sample comprising the whole analysis procedure to assess possible contamination during the procedure. The method performance was verified by analyzing the NIST SRM 1649b reference material and comparing the results with the certified concentrations of the investigated PAHs. The limits of detection (LODs) were calculated as three times the standard deviation of background peaks in the procedural blanks repeated three times. The average LOD values were 2.0 ± 0.05 ng/mL. Concentrations below the LOD were considered zero for all calculations. 

Gas chromatography coupled to mass spectrometry is a good, widely used analytical technique to assess the degree of contamination of soil and water, or the level of toxicity of waste where an assessment of chemical composition is required. It has been used in multifaceted ways in environmental and geochemical studies on various aspects of waste in scientific papers [41,43,44,45]. The method allows the identification of individual substances based on mass spectra and quantitative analysis.

## 3. Results and Discussion

### 3.1. PAH Concentrations in Soil and Combusted Solid Waste Samples from the Wild Landfill Fire

Among the PAHs of the Trzebinia samples, the following were identified (Table 2): naphthalene (N) (*m*/*z* = 128), fluorene (F) (*m*/*z* = 166), phenanthrene (P) (*m*/*z* = 178), anthracene (A) (*m*/*z* = 178), acenaphthene (Ace) (*m*/*z* = 154), acenaphthylene (Acy) (*m*/*z* = 152), fluoranthene (Fl) (*m*/*z* = 202), pyrene (Py) (*m*/*z* = 202), benzo(c)phenanthrene (BcPhe) (*m*/*z* = 252), benzo(a)anthracene (BaA) (*m*/*z* = 228), chrysene (Ch) (*m*/*z* = 228), benzo(ghi)fluoranthene (BghiFl) (*m*/*z* = 228), benzo(b + k)fluoranthene (Bb + kF)(*m*/*z* = 252), benzo(a)fluoranthene (BaF) (*m*/*z* = 252), benzo(c)fluoranthene (BcF) (*m*/*z* = 252), benzo(a)pyrene (BaP) (*m*/*z* = 252), benzo(e)pyrene (BeP) (*m*/*z* = 252), perylene (Pe) (*m*/*z* = 252), indeno[1,2,3-cd]pyrene (IP) (*m*/*z* = 276), benzo(ghi)perylene (BghiP) (*m*/*z* = 276), and dibenzo(a + h)anthracene (DB) (*m*/*z* = 278). PAHs have been reported to be among the most abundant classes of organic pollutants generated in many open burning processes [46,47]. The values of diagnostic PAH ratios in the Trzebinia samples are presented in Table 3.

In the samples analyzed, fluoranthene and pyrene were detected in 31 samples. The highest fluoranthene concentrations (ppm) were recorded in the following samples: WR6.O (6.721 ppm), WR5.O (5.559 ppm),WR1.O (4.956 ppm), WR4.O (4.873 ppm), WR2O (1.836 ppm), WR8G (1.835 ppm), WR10.O (1.457 ppm), WR5G (1.207 ppm), WR9.O (1.200 ppm), WR11.O (1.095 ppm), and WR4o (1.018 ppm), while pyrene concentrations were recorded in the following samples: WR12G (39.311 ppm), WR6o (25.577 ppm), WR5G (13.652 ppm), WR2G (12.036 ppm), WR2o (9.638 ppm), WR3G (9.113 ppm), WR5O (8.728 ppm), and WR1.O (6.552 ppm).

Phenanthrene and anthracene are among the co-extensive compounds, where the formation of anthracene is thought to result from combustion [48,49]. Lighter naphthalene (*m/z* = 128) was recorded in 24 samples: WR2G, WR1O, WR3G, WR2O, WR5G, WR6G, WR7G, WR3O, WR4O, WR8G, WR12G, WR6O, WR7O, WR1.O, WR2.O, WR3.O, WR4.O, WR5.O, WR6.O, WR8.O, WR9.O, WR10.O, WR11.O, and WR12.O. The average concentrations (ppm) of individual PAHs in the samples were as follows: N—4.305 ppm, F—0.994 ppm, P—17.455 ppm, A—0.18 ppm, Ace—6.48 ppm, Acy—4.738 ppm, Fl—1.257 ppm, Py—5.923 ppm, BcPhe—0.31 ppm, BaA—1.394 ppm, Ch—1.269 ppm, BghiFl—0.31 ppm, Bb + kF—2.056 ppm, BaF—0.533 ppm, BcF—0.396 ppm, BaP—1.504 ppm, BeP—1.318 ppm, Pe—0.295 ppm, IP—0.109 ppm, BghiP—0.545 ppm, and DB—0.32 ppm.

Some of the most abundant PAHs (except naphthalene) in controlled and uncontrolled landfill fire waste samples are reported to be phenanthrene, fluoranthene and pyrene [33]. In addition, the low-molecular weight PAHs, naphthalene, acenaphthene, acenaphthylene, anthracene, phenanthrene, and fluorene, are quickly transformed by many bacteria and fungi. Furthermore, high-molecular weight PAHs are more recalcitrant in the environment and resist both chemical and microbial biodegradation [50].

**Figure 3:**
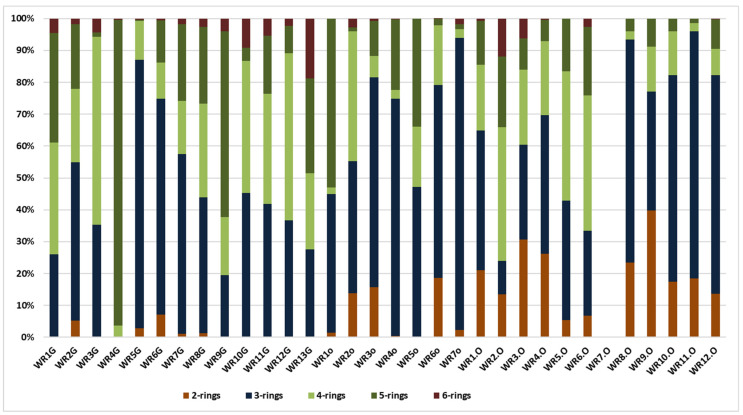
Distribution pattern (%) of 2–6-ring PAHs in the samples (ppm) from Trzebinia.

The diagram in (Figure 3) presents the distribution pattern (%) of 2–6-ring PAHs in the samples (ppm) from Trzebinia. The most dominant PAHs are 3-ring (P, A, Ace, Acy, F), which are present in 31. The sum of the 3-ring PAH values was 955.102 ppm. 4-ring PAHs (Fl, Py, BaA, Ch) represent the second value of the tested samples. These were observed in 31 samples, with a total value of 314.701 ppm. 5-ring-shaped PAHs (BghiFl, BcPhe, Bb + kF, BaF, BcF, BaP, BeP, Pe, DB) were observed in 31 samples, with a total value of 225.382 ppm. Naphthalene 2-ring PAH was observed in 25 samples, with a total value of 137.774 ppm. The lowest level of PAHs was 6-ring (IP, BghiP). These were observed in 24 samples. Naphthalene was more dominant in burnt waste than in soil samples. In contrast, 3-ring PAHs were more dominant in soil samples.

**Figure 4:**
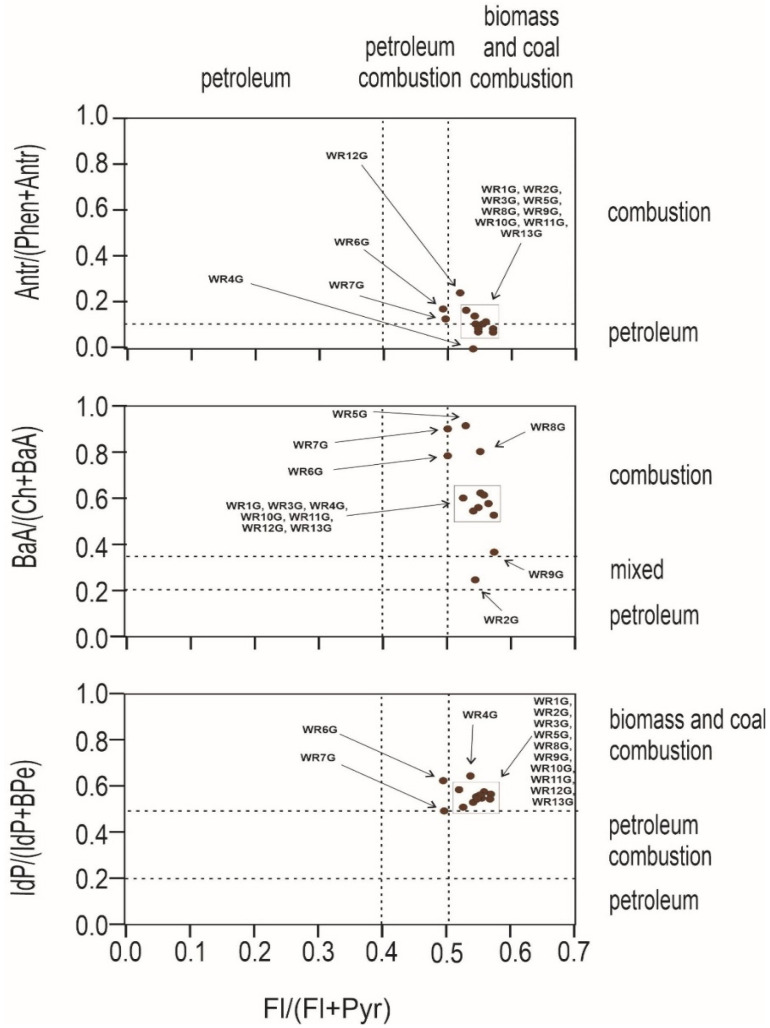
Diagnostics diagrams of PAHs in soils investigated from wild waste dump in Trzebinia on Słowackiego Street. A—anthracene, P—phenanthrene, Fl—fluoranthene, Py—pyrene, BaA—benzo(a)anthracene, Ch—chrysene, IP—indeno(1,2,3-cd)pyrene, BghiP—benzo(ghi)perylene.

**Figure 5:**
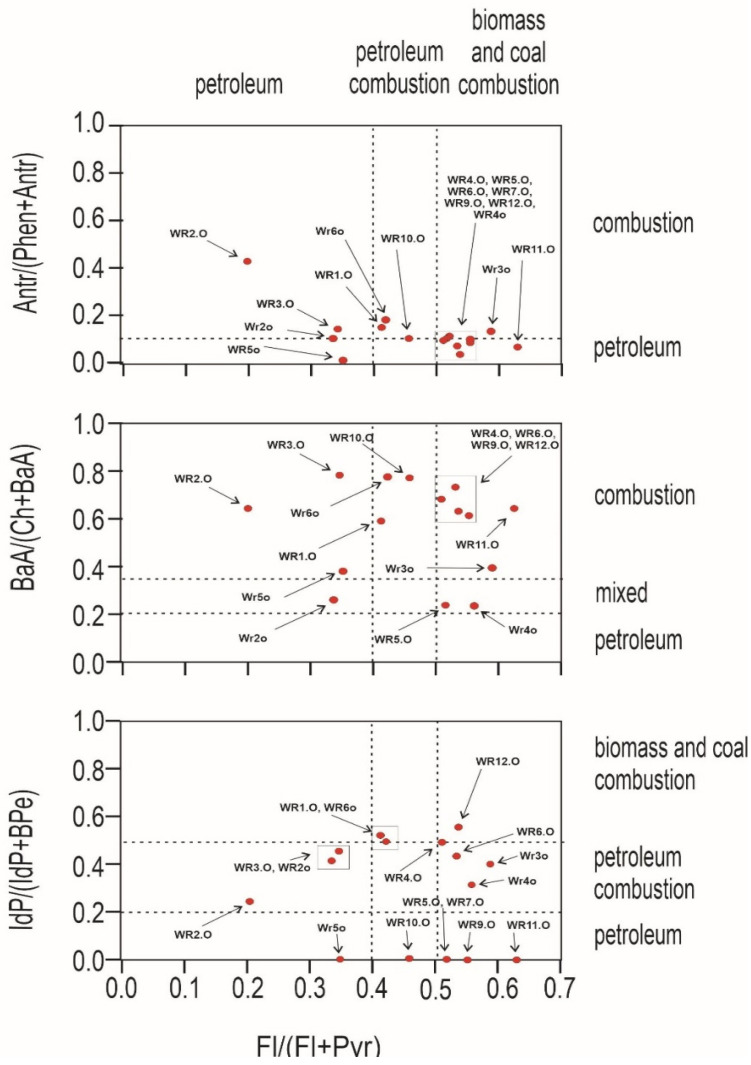
Diagnostics diagrams of PAHs in burnt waste investigated from wild waste dump in Trzebinia on Słowackiego Street. A—anthracene, P—phenanthrene, Fl—fluoranthene, Py—pyrene, BaA—benzo(a)anthracene, Ch—chrysene, IP—indeno(1,2,3-cd)pyrene, BghiP—benzo(ghi)perylene.

An important question to ask at the outset is whether the source of pollution is traffic, combustion of fossil fuels in nearby plants, or the wild landfill fire described here. For this purpose, PAH diagnostic coefficients were used (Figure 4 and Figure 5). Several PAH diagnostic ratios were calculated based on quantitative analysis (Table 3) [51]. The origins of PAHs in soil may be identified by the phenanthrene/anthracene and the fluoranthene/pyrene ratios [52]. Three types of ratios of PAHs to their possible effects were compared: anthracene and phenanthrene to fluoranthene and pyrene, benzo(a)anthracene and chrysene to fluoranthene and pyrene, and indeno[1,2,3-cd]pyrene and benzo(g,h,i)perylene to fluoranthene and pyrene. In Figure 4, the distribution of the individual soil samples is presented, whereas Figure 5 presents the distribution of burnt waste from the wild landfill in Trzebinia. The soil samples in range (A/(P + A)) were plotted in the area corresponding to combustion and petroleum to biomass and coal combustion (Fl/(Fl + Py))—WR1G, WR2G, WR3G, WR4G, WR5G, WR8G, WR9G, WR10G, WR11G, WR12G, and WR13G. In the second case, most of the samples were recorded in the combustion range (BaA/(Ch + BaA)) to biomass and coal combustion (Fl/(Fl + Py))—except the WR2G sample. In the third case, most samples—except WR6G and WR7G, were recorded, with 11 samples in the range of biomass and coal combustion IdP/(IdP + BghiP)) to biomass and coal combustion (Fl/(Fl + Py)). Samples of burnt solid waste showed high separability compared to soil samples (A/(P + A)), (BaA/(Ch + BaA)), IdP/(IdP + BghiP)) to (Fl/(Fl + Py)). The production of PAHs will presumably be controlled by waste composition (concentrations have been shown to increase dramatically when the proportion of plastic in the waste increases) and combustion conditions [53]. Escobar-Arnanz et al. [34] suggested the potential of aromatic compounds with a wide range of rings to diffuse into the environment through, among other things, emission sources, the nature of the compounds produced, and meteorological conditions, where atmospheric deposition is considered the main source of PAHs in soil [54].

The mean RTBaP concentration of the samples tested was 3.571 ppm. Above-average results were observed in 13 samples (11 soil samples, 2 burnt waste): WR1G = 5.042 ppm, WR2G = 9.366 pmm, WR3G = 5.559 pmm, WR5G = 4.127 pmm, WR6G = 5.015 ppm, WR7G = 3.783 ppm, WR8G = 5.464 ppm, WR10G = 11.134 ppm, WR11G = 7.285 ppm, WR12G = 23.513 ppm, WR13G = 5.661 ppm, WR6O = 7.894 ppm, and WR6.O = 3.886 ppm. The highest concentration was found in soil samples WR12G and WR10G. The mean MEQ concentration of the samples tested was 2.386 ppm. Above-average results were observed in 10 samples (6 soil samples, 4 burnt waste): WR1G = 3.198 ppm, WR2G = 6.211 ppm, WR3G = 4.231 ppm, WR8G = 4.107 ppm, WR10G = 3.214 ppm, WR12G = 19.836 ppm, WR2O = 2.7 ppm, WR6O = 4.766 ppm, WR1.O = 2.938 ppm, and WR6.O = 4.257 ppm. The highest concentration was found in soil sample WR12G. The TCDD-TEQ were observed in 10 samples: WR1G, WR2G, WR3G, WR8G, WR10G, WR12G, WR2O, WR6O, WR1.O, and WR6.O—the highest in soil sample WR12G = 0.05 ppm. The average ratio ∑PAH_carc_/∑PAH in the tested samples was 0.171 ppm. Above-average results were observed in 13 samples (10 soil samples, 3 burnt waste): WR1G = 0.41 ppm, WR2G = 0.234, WR3G = 0.393 ppm, WR4G = 0.44 ppm, WR8G = 0.27 ppm, WR9G = 0.391 ppm, WR10G = 0.32 ppm, WR11G = 0.33 ppm, WR12G = 0.417 ppm, WR13 = 0.353 ppm, WR2O = 0.178 ppm, WR2.O = 0.241 ppm, and WR6.O = 0.26 ppm. The highest concentration was found in soil samples WR4G and WR12G. 

Studies related to PAHs under the influence of fire in landfills have also been conducted in several types of research—[4,18,33,55].

**Figure 6:**
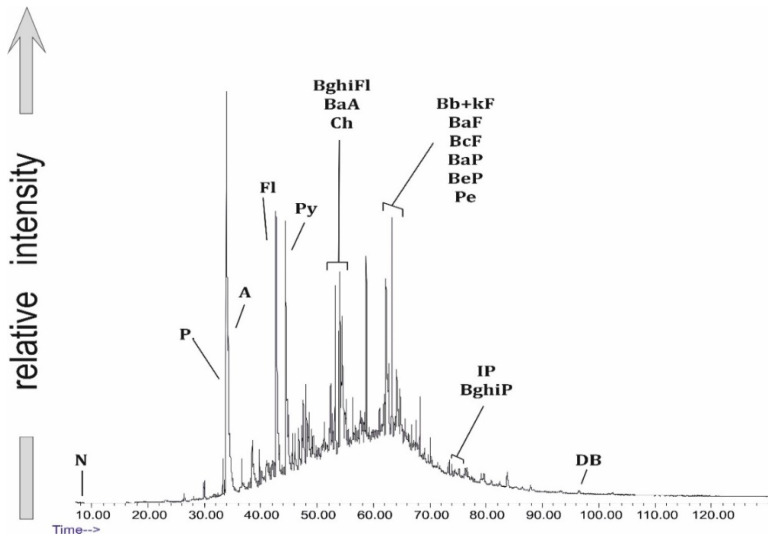
Generalized PAH distribution in sample WR12G.

The sum of PAH concentrations in the soil samples investigated shows high variability in the range of 0.964–184.611 ppm. The same conclusion can be reached for samples of combusted waste 0.000–212.045 ppm. Only sample WR7.O (unburnt wallpaper) was below the detection limit of ppm. The highest content of the PAH sum was in the burnt waste sample WR4O: 212.045 ppm. However, while 6 more samples of combusted waste were analyzed than soil samples, the mean sum of PAHs is higher in the soil samples (52.452 ppm). The samples collected may be divided into three subgroups: (i) with a maximum concentration of PAHs > 100 ppm/g in samples: WR5G, WR12G, WR4O, and WR6O (4 samples), (ii) with a mean concentration of PAHs in the range 50–100 ppm/g, including WR2G, WR6G, WR1.O, and WR11.O (4 samples), and (iii) with the lowest PAH concentrations of < 50 ppm/g, including WR1G, WR3G, WR4G, WR7G, WR8G, WR9G, WR10G, WR11G, WR13G, WR1O, WR2O, WR3O, WR5O, WR7O, WR2.O, WR3.O, WR4.O, WR5.O, WR6.O, WR7.O, WR8.O, WR9.O, WR10.O, and WR12.O (24 samples). In the samples examined, a high cumulative PAH content was found, which is alarming even years after the fire in the wild landfill. The total concentrations of all carcinogenic PAHs (BaA, BaP, Bb + kF, Ch, DB, IP) are well above the permissible values. The highest sum was found in soil samples 153.324 ppm, with the largest amount found in sample WR12G = 66.189 ppm (Figure 6). In the combusted waste samples, 59.554 ppm were measured, with the WR6O sample having the highest value of 14.056 ppm. These results may be compared with the occurrence of PAH contaminants in other parts of Poland [56,57]. In addition, it can be seen that the sum results of PAHs correspond to highly contaminated industrial soils, although there is no industrial plant around the landfill [58,59]. This also shows that soil microorganisms cannot cope with the degradation of this amount of pollutant.

To assess the effects of PAHs on health and the environmental, several indicators were calculated. They defined the toxicity of individual PAHs and the whole compound group to assess the risk caused by exposure to a PAH mixture. The reference contamination was BaP. Basically, the TEF factor for the other 16 PAHs was calculated using BaP. The toxicity value, TEQ, is the sum of individual PAH concentrations and their relative toxicity coefficients [60,61] (Table 2). The organization of carbon atoms as a bay region causes a high degree of biochemical reactivity to some PAHs and their metabolites [62]. The results showed an increased risk of carcinogenicity in the area of the fire, but no serious risk to the surrounding residents was identified. These results are comparable to other studies [33,55].

The mutagenicity equivalent MEQ and concentrations were also calculated [40]. This is one of the factors that may increase cancer risk [63]. MEQ concentrations were higher in soil samples than in combusted waste. Accordingly, WR12G recorded 19.836 ppm and WR2G 6.211 ppm. RTBaP and MEQ are quite different (RTBaP 114.26 ppm compared to MEQ 76.359 ppm). 

BaPE was used to evaluate the toxicity of PAHs (Table 2). This method is often used to calculate toxicity in soil, air, and combusted waste in this article. The values of the indicator vary considerably between the soil samples and the combusted waste. Most soil samples exceeded 1.5 ppm/g, with the highest result in sample WR12G of 15.33 ppm/g. Almost all combusted waste samples (except WR6O, WR1.O, and WR6.O) did not exceed the limit value of 1.5 ppm/g, with the highest result in sample WR6O of 4.05 ppm/g.

PAH distribution transformations occur during migration in the air, which were not observed in the source material. A group of compounds emitted on particles exhibits a different distribution from the source material due to the sorption of the pyrolytic phase itself. The formation of hydrocarbons in fires in municipal waste occurs with oxygen or with severely restricted access. It is assumed that the formation of hydrocarbon groups is different from that of pyrolysis during complete or near-complete combustion. In addition, the use of diagnostic methods obtained from the landfill fire was not considered appropriate due to anomalies of high PAH levels in soils.

2-ring naphthalenes were found to be significantly higher in combusted waste than in soil samples. This may be related to a study [64], in which, in laboratory conditions, controlled heating and burning of coal spoils was carried out to determine PAH emissions with full access and without access to oxygen. Increased 2-ring concentrations in the range of 200 °C were observed. In addition, another paper [65] established that temperatures of up to 250 °F have been measured in municipal solid waste landfills when they are undergoing a subsurface reaction. This means that a firing temperature of about 200 °C may have contributed to the increase of naphthalenes in the combusted waste.

### 3.2. MPI Concentrations in Soil and Combusted Solid Waste Samples from the Wild Landfill Fire

Methylphenanthrenes (*m*/*z* = 192) were found in 30 samples (Table 4) from the wild waste dump in Trzebinia. The average values of MPI3 and MPI1 in the samples from this set were MPI3 = 1.97 and MPI1 = 2.95. The highest MPI3 above average was recorded in samples WR1G = 1.63, WR2G = 2.01, WR4O = 3.45, WR8G = 4.34, WR9G = 3.38, WR10G = 3.27, WR5O = 4.93, WR11G = 4.39, WR13G = 3.91, and WR5.O = 2.01. On the other hand, MPI1 index above the mean was recorded in samples WR2G = 3.02, WR4O = 5.18, WR8G = 6.52, WR9G = 5.07, WR10G = 4.90, WR5O = 7.39, WR11G = 6.59, WR13G = 5.86, and WR5.O = 3.01. The mean R_c_ was 2.84. The MPI1 values were used to calculate the theoretical reflectivity value of fossil fuel vitrinite Rc based on the formula proposed by Radke [66].

R_c_ values above 2.0 correspond to a thermally highly transformed organic substance, while significant parameter variations indicate heterogeneous temperatures during the fire. At the same time, it was noted that the location of the samples taken affects the index value. In the case of soils, these high values indicate that a fire is the cause and not car traffic. An example R_c_ of car traffic and coal combustion in private fireplaces would be about 0.7–0.9 [66,67,68,69]. The mean MPI3 and MPI1 values also correspond to a high thermal transformation of the samples.

### 3.3. Compound Contents in Leachate Samples of Burnt Solid Waste from the Wild Landfill Fire

Physical and chemical results indicate that the leachates analyzed were most contaminated by major ions, namely sulfate and chloride (Table 5).

**Figure 7:**
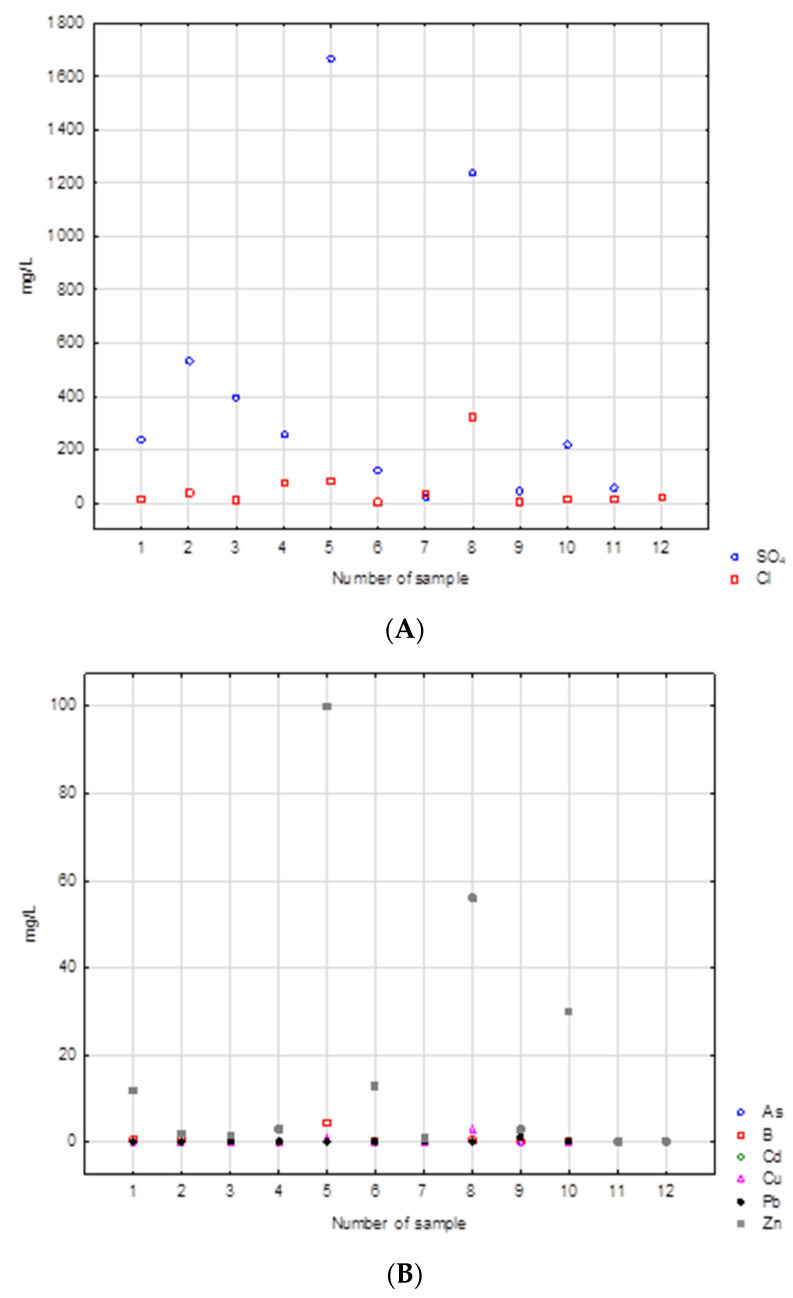
(**A**,**B**) Concentrations of main ions in the leachates analyzed.

Of the metals, zinc is the most significant contributor. The highest concentrations of sulfate were observed in samples 5 and 8. These include samples of burnt tires and rubber and foam. Both samples had sulfate contents exceeding 1000 mg/L. These samples have the highest concentrations of most contaminants. This is also true for the chloride content in these samples: 85 and 320 mg/L, respectively. Sample 8 also had a high concentration of zinc, over 50 mg/L. In this case, the highest concentration was found in sample 5 (100 mg/L). There were few arsenic, lead, cadmium, or copper concentrations in the samples tested. The concentrations of each constituent in the leachate are shown in (Figure 7A,B). The leachate produced by washing burnt rubber black waste and tires displayed a dark black color. This was most likely caused by very fine soot from the combustion process.

The results obtained from the leachate tests show that the contamination caused by metals and major ions is low compared to organic compounds (Appendix A Table A1). Similar results regarding the amount of contaminant leaching from such waste has been documented in other studies such as Hennerbert et al. [70]. Burnt tires also had significant concentrations of sulfate and zinc in the leachate, but the other parameters tested did not indicate high contamination.

The results of the physicochemical analyses were compared with the permissible values of these parameters in groundwater based on the Regulation of the Minister of Maritime Economy and Inland Navigation of 11 October 2019, on the criteria and method of assessing the state of groundwater bodies. In the case of sulfates, three samples (WR2.O, WR5.O, and WR8.O) would be classified as water quality class V (poor chemical status). Samples WR7.O, WR9.O, and WR12.O would be classified as quality class I (good chemical condition). It should be noted that the V class of water quality includes water samples with sulfate concentrations higher than 500 mg/L. In this context, samples with concentrations exceeding 1200 mg/L seem particularly dangerous. Typical sulfate levels in fresh water range from 0 to 630 mg/L in rivers, from 2 to 250 mg/L in lakes and from 0 to 230 mg/L in groundwater [71].

It is assumed that the chloride concentration in unpolluted waters can reach 10 mg/L. The concentration of this component in precipitation fluctuates around 1 mg/L. The high chloride content in waters and soils is a result of anthropogenic activity. It is also a typical indicator of groundwater pollution in the area of landfills [72]. The upper limit of the hydrochemical background for this component is 60 mg/L. In the case of sample numbers WR4.O, WR5.O, and WR8.O, these values are much higher. In the last one, it would be classified as water quality class IV. The high chloride content of this sample may come from dyes in the textiles.

In the case of arsenic, the acceptable concentration value for the first class is 0.01 mg/L. On the other hand, the upper limit of the natural hydrochemical background is twice as high. The arsenic content in the samples tested ranges from 0.0062 (WR10.O) to 0.085 mg/L (WR3.O). This suggests that the most contaminated samples would be classified in quality class IV.

Only two samples (WR4.O and WR5.O) have the content of boron ions appropriate for the V quality class. In other cases, these are concentrations characteristic of quality class III and higher. Boron is an important pollution indicator for municipal waste, but it is also a component of paints.

In the event of contamination with copper, cadmium, or lead, most samples would be graded into third and fourth quality classes. However, there are also samples whose concentration of the mentioned components suggests that they belong to class V. For example, the concentration of lead in sample WR9.O is ten times higher than the lower limit for class V, and in the case of copper ions, a concentration six times higher was found in sample WR8.O.

In the case of zinc ions, the natural hydrochemical background is in the range 0.005–0.050 mg/L. Only two of the samples tested could be classified as class II of water quality. Most, however, are classified in the fourth and fifth quality class. For samples WR10.O and WR5.O, the concentrations of zinc ions are 15 times and 50 times higher, respectively, than the fifth-class limit. It should be borne in mind that high zinc contents in the waste tested may be, among other things, the effect of the use of zinc oxide in tire vulcanization.

The results from the laboratory were also compared with the acceptable standards for leaching pollutants specified in the Regulation of the Minister of Economy of July 16, 2015, on allowing waste to be deposited in landfills [73]. The values were converted depending on the size of the waste sample collected. For all samples tested, except for sample number 3 (sponge), the limit values were reduced by 10 times, and for this sample by 20. When comparing the results obtained with the standards, for all parameters, except for lead and zinc, the requirements for depositing non-municipal waste are met. In the case of lead, only one sample (WR9.O) slightly exceeds the allowed concentration. The situation is much worse with zinc. For this sample size, the acceptable value limit is 5 mg/L. In the set of analyses obtained, as many as 5 samples exceed this value. One of the samples (WR5.O) was up to 20 times higher. It should also be noted that these are the results of leachability tests obtained from small samples of waste. These values should be properly recalculated depending on the total mass of waste located in the wild landfill.

It is also possible to constantly monitor changes in soil or water quality in the vicinity of such facilities with the use of sensors and artificial intelligence [74,75].

The concentrations of the individual components are not too high compared to the typical values that can be recorded in soils. A similar lack of increased concentration of inorganic compounds in soils in the vicinity of the fires was also observed in other studies [55,76,77].

## 4. Conclusions

Soil samples and incinerated waste were analyzed to determine how a wild landfill fire may affect the environment and the health of the surrounding inhabitants. The most significant finding of the research is that the currently deposited burnt solid waste in the wild landfill poses a potentially permanent hazard to the environment. Samples taken even a long time after the fire started in the study area contain significant concentrations of hazardous organic compounds, in particular PAHs. This includes both soil samples and solid waste transformed by the fire. The results obtained show a high total concentration of the PAHs found in the samples. These results show that these soils are at similar pollution levels to soils heavily polluted by industry, although there are no industrial installations on the site itself or in the surrounding area. However, lower levels of mutagenicity and carcinogenicity were observed. ΣPAH_carc_/ΣPAH did not approach 1, which is considered a potential health risk, in any of the samples tested. However, since the general summary PAH level is high, it follows that carcinogenicity is also high. 

No significant spikes in heavy metal content were observed in leachate collected from the incinerated waste samples. This may be justified because, during the incineration of solid waste such as tires and black rubber, more organic compounds are formed by high temperature and oxidation.

Personal protective measures (gloves, protective clothing, and protective masks) are required for workers in all future work on the site of a burnt wild landfill. They aim to reduce skin contact with dust and reduce the risk of contaminated dust particles being breathed into the body, which could increase the risk of lung cancer.

Research of this type must be carried out in conjunction with environmental monitoring to identify potential threats to the environment and human health early. This is important since the amount of illegal waste stored in previously unsecured and unattended places is growing year by year.

It is worth mentioning that similar tests are currently being carried out at sites after fires in Sosnowiec and Sobolew to obtain a larger database of results for contaminant tests.

In addition, future research could be complemented by the development of a pollutant migration model and geochemical modeling using appropriate programs.

## Figures and Tables

**Table 1:** Sample code and description.

Sample	Description of the Sample	Sample	Description of the Sample
WR1G	soil sample	WR12G	soil sample
WR2G	soil sample	WR6O	burnt waste sample
WR3G	soil sample	WR7O	burnt waste sample
WR1O	burnt waste sample	WR13G	soil sample
WR4G	soil sample	WR1.O	burnt tires
WR2O	burnt waste sample	WR2.O	burnt rubber black waste
WR5G	soil sample	WR3.O	burnt rubber black waste and brown sponge
WR6G	soil sample	WR4.O	burnt rubber black waste
WR7G	soil sample	WR5.O	burnt rubber black waste
WR8G	soil sample	WR6.O	burnt rubber black waste
WR3O	burnt waste sample	WR7.O	unburnt wallpaper
WR4O	burnt waste sample	WR8.O	burnt textiles
WR9G	soil sample	WR9.O	burnt textiles
WR10G	soil sample	WR10.O	burnt tires
WR11G	soil sample	WR11.O	burnt textiles
WR5O	burnt waste sample	WR12.O	burnt textiles

**Table 2:** Polycyclic aromatic hydrocarbon concentrations in the Trzebinia samples (ppm).

**Sample**	**N**	**F**	**P**	**A**	**Ace**	**Acy**	**Fl**	**Py**	**BcPhe**	**BaA**	**Ch**	**BghiFl**	**Bb + kF**	**BaF**
WR1G	-	0.078	6.321	0.018	0.007	0.374	0.184	5.338	0.067	1.599	2.053	0.067	2.902	0.862
WR2G	5.045	0.076	33.445	0.025	4.247	9.319	0.427	12.036	0.267	3.628	5.658	0.267	6.053	1.360
WR1O	0.231	0.554	2.937	0.006	2.884	0.747	0.011	0.019	0.021	0.048	0.265	0.021	0.162	0.019
WR3G	0.002	0.045	8.766	0.042	0.002	0.019	0.464	9.113	0.231	2.469	2.754	0.231	4.085	1.281
WR4G	-	-	-	0.003	-	-	0.116	0.300	0.007	0.040	0.047	0.007	0.139	0.049
WR2O	4.740	0.913	6.562	0.201	0.166	6.442	1.836	9.638	0.920	0.522	2.015	0.920	2.295	0.773
WR5G	4.205	10.960	61.047	0.464	14.697	35.399	1.207	13.652	1.309	2.672	0.329	1.309	2.009	0.559
WR6G	4.700	3.441	27.542	0.180	7.209	6.462	0.255	5.421	0.739	1.367	0.512	0.739	1.831	0.379
WR7G	0.365	0.950	15.880	0.078	0.643	2.452	0.241	4.453	0.539	1.004	0.155	0.539	1.693	0.398
WR3O	9.310	2.769	24.653	0.202	4.949	6.208	0.793	2.661	0.009	0.163	0.338	0.009	0.113	0.040
WR4O	1.306	0.246	96.113	0.400	39.065	65.201	1.018	5.655	0.344	0.170	0.758	0.344	0.991	0.134
WR8G	0.406	0.537	11.149	0.084	0.240	1.388	1.835	5.954	0.629	1.049	0.355	0.629	3.401	0.890
WR9G	-	0.026	1.966	0.004	0.008	0.433	0.099	1.327	0.020	0.241	0.586	0.020	0.887	0.280
WR10G	-	0.379	7.225	0.030	0.014	0.392	0.636	5.407	0.084	0.712	0.643	0.084	2.004	0.666
WR5O	-	-	7.866	0.043	1.028	15.028	0.413	8.728	0.037	0.163	0.369	0.037	0.126	0.021
WR11G	-	0.115	5.749	0.006	0.327	0.690	0.299	3.852	0.088	0.764	0.800	0.088	1.744	0.588
WR12G	0.166	0.380	48.182	0.243	0.191	0.936	0.682	39.311	1.131	16.252	15.125	1.131	20.696	3.741
WR6O	31.277	7.159	74.291	0.017	20.191	0.049	0.164	25.577	1.956	4.122	1.567	1.956	4.390	0.704
WR13G	-	-	2.561	0.007	0.025	-	0.239	1.357	0.029	0.338	0.291	0.029	0.748	0.257
WR7O	0.659	3.180	21.739	0.032	1.298	0.075	0.074	0.063	0.011	0.505	0.126	0.011	0.188	0.059
WR1.O	13.455	-	20.564	1.125	6.286	-	4.956	6.552	0.791	0.880	0.868	0.791	1.969	0.572
WR2.O	1.743	-	0.827	0.189	0.352	-	0.886	3.250	0.078	0.354	0.920	0.078	0.538	0.377
WR3.O	3.945	-	2.434	0.130	1.265	-	0.826	1.454	0.028	0.552	0.212	0.028	0.362	0.098
WR4.O	12.840	-	16.321	0.591	4.407	-	4.873	4.355	0.055	1.276	0.836	0.055	0.654	0.346
WR5.O	1.539	-	9.871	0.403	0.610	-	5.559	4.802	0.412	0.256	1.125	0.412	1.493	0.209
WR6.O	2.448	-	9.387	0.250	-	-	6.721	5.475	0.039	2.100	1.079	0.039	2.200	0.625
WR7.O	-	-	-	-	-	-	-	-	-	-	-	-	-	-
WR8.O	7.943	-	4.683	0.073	18.866	-	0.565	0.071	0.013	0.199	0.043	0.013	0.596	0.194
WR9.O	6.929	-	3.774	0.123	2.605	-	1.200	0.902	0.020	0.199	0.176	0.020	0.590	0.262
WR10.O	4.557	-	10.589	0.411	6.042	-	1.457	1.599	0.007	0.400	0.165	0.007	0.307	0.222
WR11.O	16.953	-	11.960	0.300	58.733	-	1.095	0.602	0.044	0.331	0.258	0.044	0.345	0.367
WR12.O	3.012	-	4.173	0.063	11.002	-	0.783	0.629	0.010	0.237	0.193	0.010	0.284	0.732
**Sample**	**BcF**	**BaP**	**BeP**	**Pe**	**IP**	**BghiP**	**DB**	**PAH sum ppm/g**	**PAH sum μg/kg**	**RTBaP**	**∑PAH_carc_/∑PAH**	**MEQ**	**TCDD-TEQ**	**BaPE ppm/g**
WR1G	0.347	1.908	1.964	0.407	0.239	0.931	0.507	26.174	26,173.7	5.042	0.410	3.198	0.01	2.53
WR2G	1.093	3.670	4.636	1.005	0.323	1.428	0.886	89.850	89,850.1	9.366	0.234	6.211	0.02	4.87
WR1O	0.020	0.049	0.033	-	-	-	-	7.795	7795.0	0.080	0.066	0.098	-	0.06
WR3G	0.715	2.602	3.026	0.508	0.203	0.899	0.429	37.884	37,883.9	5.559	0.393	4.231	0.01	3.31
WR4G	0.005	0.116	0.086	0.005	0.012	0.032	-	0.964	963.9	0.139	0.440	0.164	-	0.13
WR2O	0.783	1.844	0.794	0.440	0.117	0.869	-	38.050	38,049.8	2.277	0.178	2.700	0.01	2.05
WR5G	0.322	1.322	1.082	0.188	0.104	0.502	0.425	149.558	149,557.7	4.127	0.046	2.320	-	1.89
WR6G	0.319	1.501	1.242	0.235	0.085	0.261	0.620	60.340	60,340.4	5.015	0.096	2.339	-	2.09
WR7G	0.346	1.514	1.516	0.341	0.103	0.532	0.382	33.757	33,757.3	3.783	0.159	2.267	-	1.93
WR3O	0.020	0.149	0.164	0.022	0.052	0.400	0.194	43.909	43,908.8	1.247	0.019	0.348	-	0.29
WR4O	0.092	0.085	0.814	0.182	0.035	0.398	-	212.045	212,045.2	0.465	0.010	0.483	-	0.17
WR8G	0.431	2.873	1.976	0.447	0.158	0.650	0.408	35.083	35,082.6	5.464	0.270	4.107	0.01	3.43
WR9G	0.091	0.619	0.506	0.065	0.094	0.399	0.310	7.981	7981.2	2.339	0.391	1.065	-	0.89
WR10G	0.346	1.780	1.761	0.170	0.317	1.313	1.780	25.744	25,743.7	11.134	0.320	3.214	0.01	3.06
WR5O	0.010	0.079	0.033	0.039	-	-	-	34.022	34,021.9	0.145	0.022	0.139	-	0.10
WR11G	0.209	1.144	1.262	0.170	0.188	0.711	1.156	19.950	19,949.6	7.285	0.330	2.186	-	2.02
WR12G	4.335	11.982	12.421	3.330	0.676	2.407	1.457	184.611	184,611.0	23.513	0.417	19.836	0.05	15.33
WR6O	0.934	3.024	2.988	0.508	0.187	-	0.765	150.549	150,549.3	7.894	0.081	4.766	0.01	4.05
WR13G	0.118	0.727	0.516	0.053	0.135	1.623	0.928	9.981	9981.1	5.661	0.353	1.566	-	1.37
WR7O	0.029	0.087	0.021	0.037	-	0.530	-	28.065	28,064.6	0.238	0.032	0.278	-	0.13
WR1.O	0.515	2.260	1.590	0.273	0.082	0.384	-	50.458	50,458.1	2.664	0.102	2.938	0.01	2.46
WR2.O	0.257	0.853	0.574	0.116	0.090	1.450	-	11.190	11,189.7	1.115	0.241	1.336	-	0.92
WR3.O	0.064	0.357	0.231	0.090	0.112	0.684	-	8.927	8927.3	0.541	0.129	0.661	-	0.42
WR4.O	0.235	1.322	0.582	0.061	0.033	0.172	-	36.172	36,172.4	1.592	0.086	1.647	-	1.45
WR5.O	0.281	1.379	0.136	0.455	-	-	-	27.402	27,402.3	1.592	0.157	1.792	-	1.50
WR6.O	0.324	3.325	1.058	0.198	0.121	0.809	-	33.750	33,750.0	3.886	0.260	4.257	0.01	3.62
WR7.O	-	-	-	-	-	-	-	-	-	-	-	-	-	-
WR8.O	0.058	0.232	0.260	-	-	-	-	25.866	25,866.4	0.345	0.032	0.398	-	0.29
WR9.O	-	0.350	0.283	-	-	-	-	10.504	10,503.9	0.447	0.078	0.516	-	0.40
WR10.O	0.139	0.212	0.147	-	-	-	-	21.705	21,705.1	0.313	0.042	0.325	-	0.26
WR11.O	0.159	0.223	0.177	-	-	-	-	74.640	74,639.7	0.386	0.013	0.341	-	0.27
WR12.O	0.094	0.526	0.292	0.089	0.012	0.047	-	19.174	19,173.9	0.606	0.060	0.632	-	0.56
WR6O	0.347	1.908	1.964	0.407	0.239	0.931	0.507	26.174	26,173.7	5.042	0.410	3.198	0.01	2.53
WR13G	1.093	3.670	4.636	1.005	0.323	1.428	0.886	89.850	89,850.1	9.366	0.234	6.211	0.02	4.87
WR7O	0.020	0.049	0.033	-	-	-	-	7.795	7795.0	0.080	0.066	0.098	-	0.06
WR1.O	0.715	2.602	3.026	0.508	0.203	0.899	0.429	37.884	37,883.9	5.559	0.393	4.231	0.01	3.31
WR2.O	0.005	0.116	0.086	0.005	0.012	0.032	-	0.964	963.9	0.139	0.440	0.164	-	0.13
WR3.O	0.783	1.844	0.794	0.440	0.117	0.869	-	38.050	38,049.8	2.277	0.178	2.700	0.01	2.05
WR4.O	0.322	1.322	1.082	0.188	0.104	0.502	0.425	149.558	149,557.7	4.127	0.046	2.320	-	1.89
WR5.O	0.319	1.501	1.242	0.235	0.085	0.261	0.620	60.340	60,340.4	5.015	0.096	2.339	-	2.09
WR6.O	0.346	1.514	1.516	0.341	0.103	0.532	0.382	33.757	33,757.3	3.783	0.159	2.267	-	1.93
WR7.O	0.020	0.149	0.164	0.022	0.052	0.400	0.194	43.909	43,908.8	1.247	0.019	0.348	-	0.29
WR8.O	0.092	0.085	0.814	0.182	0.035	0.398	-	212.045	21,2045.2	0.465	0.010	0.483	-	0.17
WR9.O	0.431	2.873	1.976	0.447	0.158	0.650	0.408	35.083	35,082.6	5.464	0.270	4.107	0.01	3.43
WR10.O	0.091	0.619	0.506	0.065	0.094	0.399	0.310	7.981	7981.2	2.339	0.391	1.065	-	0.89
WR11.O	0.346	1.780	1.761	0.170	0.317	1.313	1.780	25.744	25,743.7	11.134	0.320	3.214	0.01	3.06
WR12.O	0.010	0.079	0.033	0.039	-	-	-	34.022	34,021.9	0.145	0.022	0.139	-	0.10

N—naphthalene, F—fluorene, P—phenanthrene, A—anthracene, Ace—acenaphthene, Acy—acenaphthylene, Fl—fluoranthene, Py—pyrene, BcPhe—benzo(c)phenanthrene, BaA—benzo(a)anthracene, Ch—chrysene, BghiFl—benzo(ghi)fluoranthene, Bb + kF—benzo(b + k) fluoranthene, BaF—benzo(a)fluoranthene, BcF—benzo(c)fluoranthene, BaP—benzo(a)pyrene, BeP—benzo(e)pyrene, Pe—perylene, IP—indeno[1,2,3-cd]pyrene, BghiP—benzo(ghi)perylene, DB—dibenzo(a + h)anthracene.

**Table 3:** Values of diagnostic PAH ratios in the Trzebinia samples.

Sample	P/A	A/P	A/(A + P)	Fl/(Fl + Py)	Fl/Py	Fl/(Fl + P)	BaA/(BaA + Ch)	BaP/BghiP	IP/BghiP	IP/(IP + BghiP)	BaA/BaP	Py/BaP
WR1G	10.62	0.09	0.09	0.57	1.32	0.81	0.53	2.45	1.31	0.57	1.34	4.98
WR2G	6.12	0.16	0.14	0.54	1.19	0.62	0.24	3.08	1.15	0.54	1.58	5.83
WR3G	15.70	0.06	0.06	0.87	6.85	0.14	0.21	-	-	-	1.58	0.68
WR1O	8.04	0.12	0.11	0.54	1.19	0.82	0.56	3.46	1.15	0.53	1.52	6.24
WR4G	-	-	-	0.54	1.16	1.00	0.55	4.34	1.83	0.65	0.56	4.61
WR2O	8.22	0.12	0.11	0.34	0.51	0.74	0.27	2.53	0.69	0.41	0.45	9.31
WR5G	4.93	0.20	0.17	0.53	1.12	0.49	0.92	3.15	1.06	0.51	3.25	18.39
WR6G	4.79	0.21	0.17	0.50	0.98	0.42	0.79	6.86	1.66	0.62	1.46	6.43
WR7G	6.59	0.15	0.13	0.50	0.99	0.51	0.90	3.40	0.98	0.50	1.06	5.24
WR8G	6.21	0.16	0.14	0.59	1.43	0.37	0.41	0.44	0.66	0.40	1.76	31.85
WR3O	7.80	0.13	0.11	0.56	1.27	0.22	0.24	0.26	0.45	0.31	3.20	118.12
WR4O	12.42	0.08	0.07	0.55	1.22	0.71	0.81	5.28	1.24	0.55	0.59	3.69
WR9G	14.12	0.07	0.07	0.57	1.32	0.77	0.37	1.85	1.20	0.55	0.63	3.82
WR10G	8.73	0.11	0.10	0.55	1.24	0.78	0.61	1.62	1.23	0.55	0.64	5.41
WR11G	59.77	0.02	0.02	0.35	0.54	0.70	0.39	-	-	-	3.31	196.45
WR5O	8.57	0.12	0.10	0.56	1.28	0.76	0.58	1.92	1.35	0.57	1.07	6.00
WR12G	3.08	0.33	0.25	0.52	1.09	0.77	0.61	5.98	1.43	0.59	2.17	5.81
WR6O	4.30	0.23	0.19	0.42	0.73	0.49	0.79	3.73	0.98	0.50	2.19	15.05
WR7O	11.39	0.09	0.08	0.55	1.22	0.71	0.62	1.50	1.19	0.54	0.75	3.33
WR13G	22.52	0.04	0.04	0.95	20.53	0.18	0.85	-	-	-	9.32	1.28
WR1.O	5.39	0.19	0.16	0.41	0.71	0.46	0.59	1.35	1.09	0.52	3.27	26.97
WR2.O	1.29	0.77	0.44	0.20	0.25	0.79	0.65	0	0.32	0.24	-	-
WR3.O	5.53	0.18	0.15	0.35	0.53	0.55	0.79	0.62	0.84	0.46	2.49	7.26
WR4.O	8.14	0.12	0.11	0.51	1.04	0.51	0.69	9.19	0.98	0.49	1.55	5.87
WR5.O	7.23	0.14	0.12	0.52	1.08	0.67	0.24	-	-	-	0.30	6.20
WR6.O	11.06	0.09	0.08	0.53	1.14	0.72	0.74	4.91	0.76	0.43	1.01	2.93
WR7.O	7.74	0.13	0.11	0.52	1.08	0.47	-	-	-	-	-	-
WR8.O	18.92	0.05	0.05	0.88	7.45	0.30	0.87	-	-	-	1.38	0.54
WR9.O	9.05	0.11	0.10	0.55	1.24	0.53	0.62	-	-	-	0.92	4.60
WR10.O	7.60	0.13	0.12	0.46	0.85	0.33	0.78	-	-	-	3.02	13.41
WR11.O	11.74	0.09	0.08	0.63	1.70	0.24	0.65	-	-	-	2.38	4.80
WR12.O	19.63	0.05	0.05	0.54	1.16	0.40	0.64	13.31	1.25	0.56	0.72	2.13

P—phenanthrene, A—anthracene, Fl—fluoranthene, Py—pyrene, BaA—benzo(a)anthracene, Ch—chrysene, BaP—benzo(a)pyrene, BeP—benzo(e)pyrene, Pe—perylene, IP—indeno(1,2,3-cd)pyrene, BghiP—benzo(ghi)perylene.

**Table 4:** Values of geochemical ratios found in DEP extracts ratios in the Trzebinia samples.

Sample	MPI3 ^(1)^	MPI1 ^(2)^	R_c_ ^(3)^	Sample	MPI3 ^(1)^	MPI1 ^(2)^	R_c_ ^(3)^
WR1G	1.63	2.45	2.61	WR12G	0.46	0.69	1.02
WR2G	2.01	3.02	3.12	WR6O	1.02	1.53	1.78
WR1O	1.23	1.85	2.06	WR13G	3.91	5.86	5.68
WR3G	1.16	1.75	1.97	WR7O	1.89	2.83	2.95
WR2O	1.07	1.60	1.84	WR1.O	0.93	1.40	1.66
WR5G	1.86	2.78	2.90	WR2.O	0.74	1.10	1.39
WR6G	1.44	2.16	2.35	WR3.O	1.29	1.93	2.14
WR7G	1.29	1.93	2.14	WR4.O	1.46	2.19	2.37
WR3O	1.65	2.48	2.63	WR5.O	2.01	3.01	3.11
WR4O	3.45	5.18	5.06	WR6.O	1.89	2.84	2.95
WR8G	4.34	6.52	6.26	WR8.O	1.65	2.47	2.62
WR9G	3.38	5.07	4.96	WR9.O	0.69	1.03	1.33
WR10G	3.27	4.90	4.81	WR10.O	1.27	1.90	2.11
WR5O	4.93	7.39	7.05	WR11.O	1.79	2.68	2.81
WR11G	4.39	6.59	6.33	WR12.O	0.90	1.35	1.61

^(1)^ MPI-3 = (2-methylphenanthrene + 3-methylphenathrene)/(1-methylphenathrene + 9-methylphenathrene); *m*/*z* = 192; thermal maturity [67]. ^(2)^ MPI-1 = 1.5(2-methylphenanthrene + 3-methylphenanthrene)/(phenanthrene + 1-methylphenanthrene + 9 methylphenanthrene); thermal maturity [66]. ^(3)^ R_c_ = 0.60 MPI-1 + 0.40; calculated according to the [67] formula.

**Table 5:** Results of physicochemical analyses of the leachates analyzed.

Ions [mg/L]	Mean	Min	Max	St. Dev
SO_4_	401.367	17.900	1670.00	524.607
Cl	51.881	0.7700	320.00	88.540
As	0.032	0.0062	0.09	0.031
B	0.960	0.0770	4.60	1.430
Cd	0.008	0.0001	0.04	0.012
Cu	0.359	0.0049	3.00	0.857
Pb	0.108	0.0067	1.10	0.312
Zn	18.468	0.2200	100.00	30.570

## Nomenclature

A—anthracene	Ace—acenaphthene
Acy—acenaphthylene	BaA—benzo(a)anthracene
BaF—benzo(a)fluoranthene	BaP—benzo(a)pyrene
Bb + kF—benzo(b + k)fluoranthene	BcF—benzo(c)fluoranthene
BcPhe—benzo(c)phenanthrene	BeP—benzo(e)pyrene
BghiFl—benzo(ghi)fluoranthene	BghiP—benzo(ghi)perylene
Ch—chrysene	DB—dibenzo(a + h)anthracene
DMR—Dimethylnaphthalene Ratio	Fl—fluoranthene
F—fluorene	GC-MS—Gas chromatography-mass spectrometry
IP—indeno[1,2,3-cd]pyrene	ICP-OES—Inductively Coupled Plasma Optical Emission Spectrometry
MNR—Methylnaphthalene Ratio	MOE—Ministry of Environment
MPI—Methylphenanthrene Index	MSW—Municipal solid waste
N—naphthalene	P—phenanthrene
PAHs—Polycyclic aromatic hydrocarbons	Pe—perylene
Py—pyrene	TEF—Toxicity Equivalence Factor
TEQ—Toxic Equivalent	TNR—Trimethylnaphthalene Ratio

## Appendix A

**Table A1:** **Results of** chemical analyses.

	Unit	SO_4_ [mg/L]	Cl [mg/L]	As [µg/L]	B [mg/l]	Cd [µg/L]	Cu [µg/L]	Pb [µg/L]	Zn [mg/L]
No.									
WR1.O		239.0	13.0	64.0	0.6	28.0	12.0	12.0	12.0
WR2.O		534.0	38.0	82.0	1.0	0.1	4.9	13.0	1.9
WR3.O		397.0	8.1	85.0	1.1	0.4	70.0	15.0	1.6
WR4.O		255.0	76.0	59.0	3.1	1.5	50.0	9.6	2.9
WR5.O		1670.0	83.0	9.0	4.6	18.0	760.0	41.0	100.0
WR6.O		121.0	0.8	7.0	0.1	27.0	83.0	30.0	13.0
WR7.O		20.8	30.0	7.0	0.1	0.2	150.0	12.0	0.85
WR8.O		1240.0	320.0	14.0	0.4	35.0	300.0	13.0	56.0
WR9.O		43.5	2.7	8.0	0.1	4.6	37.0	110.0	2.9
WR10.O		220.0	16.0	6.0	0.2	.5	68.0	16.0	30.0
WR11.O		58.2	15.0	10.0	0.1	0.1	20.0	6.7	0.22
WR12.O		17.9	20.0	35.0	0.1	0.9	59.0	33.0	0.25
